# Metagenomic investigation of the microbial diversity in a chrysotile asbestos mine pit pond, Lowell, Vermont, USA

**DOI:** 10.1016/j.gdata.2016.11.004

**Published:** 2016-11-05

**Authors:** Heather E. Driscoll, James J. Vincent, Erika L. English, Elizabeth D. Dolci

**Affiliations:** a Vermont Genetics Network, Department of Biology, Norwich University, 158 Harmon Drive, Northfield, VT 05663, USA; b Vermont Genetics Network, Department of Biology, University of Vermont, 337 Marsh Life Science, Burlington, VT 05405, USA; c Department of Environmental and Health Sciences, Johnson State College, 337 College Road, Johnson, VT 05656, USA

**Keywords:** whole metagenome shotgun sequencing, Illumina, serpentine, asbestos, alkaline, metagenomic analysis, microbial diversity

## Abstract

Here we report on a metagenomics investigation of the microbial diversity in a serpentine-hosted aquatic habitat created by chrysotile asbestos mining activity at the Vermont Asbestos Group (VAG) Mine in northern Vermont, USA. The now-abandoned VAG Mine on Belvidere Mountain in the towns of Eden and Lowell includes three open-pit quarries, a flooded pit, mill buildings, roads, and > 26 million metric tons of eroding mine waste that contribute alkaline mine drainage to the surrounding watershed. Metagenomes and water chemistry originated from aquatic samples taken at three depths (0.5 m, 3.5 m, and 25 m) along the water column at three distinct, offshore sites within the mine's flooded pit (near 44°46′00.7673″, − 72°31′36.2699″; UTM NAD 83 Zone 18 T 0695720 E, 4960030 N). Whole metagenome shotgun Illumina paired-end sequences were quality trimmed and analyzed based on a translated nucleotide search of NCBI-NR protein database and lowest common ancestor taxonomic assignments. Our results show strata within the pit pond water column can be distinguished by taxonomic composition and distribution, pH, temperature, conductivity, light intensity, and concentrations of dissolved oxygen. At the phylum level, metagenomes from 0.5 m and 3.5 m contained a similar distribution of taxa and were dominated by Actinobacteria (46% and 53% of reads, respectively), Proteobacteria (45% and 38%, respectively), and Bacteroidetes (7% in both). The metagenomes from 25 m showed a greater diversity of phyla and a different distribution of reads than the two upper strata: Proteobacteria (60%), Actinobacteria (18%), Planctomycetes, (10%), Bacteroidetes (5%) and Cyanobacteria (2.5%), Armatimonadetes (< 1%), Verrucomicrobia (< 1%), Firmicutes (< 1%), and Nitrospirae (< 1%). Raw metagenome sequence data from each sample reside in NCBI's Short Read Archive (SRA ID: SRP056095) and are accessible through NCBI BioProject PRJNA277916.

**Table:** 

Specifications	
Organism/cell line/tissue	*Mine drainage metagenome*
Sex	*Not applicable*
Sequencer or array type	*Illumina HiSeq 2000*
Data format	*Raw data: FASTQ files*
Experimental factors	*Environmental samples*
Experimental features	*Whole metagenome shotgun sequencing followed by taxonomic profiling using RAPSearch2, MEGAN, and STAMP*
Consent	*Not applicable*
Sample source location	*Eight aquatic samples from three adjacent sites, Lowell Mine it Pond, Vermont Asbestos Group Mine, Lowell, Vermont, USA (44°46′00.7673″, − 72°31′36.2699″; UTM NAD 83 Zone 18 T, 0695720 E, 4960030 N)*

## 1 Direct link to deposited data

NCBI BioProject PRJNA277916 includes raw data and sample metadata, including water chemistry: http://www.ncbi.nlm.nih.gov/bioproject/PRJNA277916.

## 2 Experimental Design, Materials and Methods

### 2.1 Study site description

**Fig. 1:**
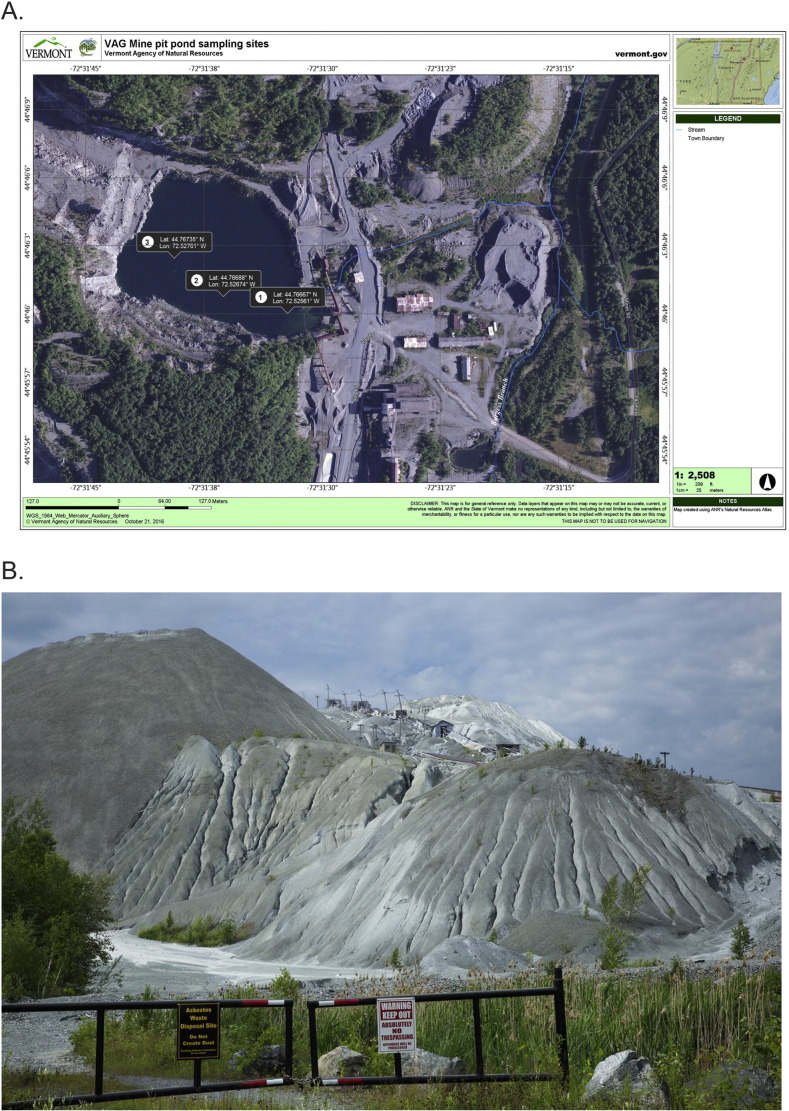
Vermont Asbestos Group Mine area: A) Aerial map with sample collection sites and Burgess Branch stream colored blue (map source: http://anrmaps.vermont.gov/websites/anra5/). B) Lowell tailings piles adjacent to the pit pond and sample collection site (photo source: Steve Schlipf, steveschlipfphotography.com).

The Vermont Asbestos Group (VAG) Mine, situated on a chrysotile asbestos-bearing ultramafic rock outcrop located within the Lamoille and Missisquoi River watersheds in northern Vermont, USA, is an abandoned asbestos mine and mill that ceased operation in 1993. High levels of chrysotile asbestos fibers and elevated concentrations of magnesium, nickel, chromium, and arsenic have been recorded at several aquatic and terrestrial sites within the VAG Mine area [1]. The site of sample collection within the VAG Mine area was the Lowell mine pit pond, a flooded open pit quarry adjacent to massive tailings piles (Fig. 1). The mineralogical character of the mine waste was identified previously (Mg, Si, Fe, and Al, major constituents; Ni and Cr, minor constituents) [2]. The water column of the VAG mine's pit pond was sampled at three sites [site (1) UTM NAD 83 Zone 18 T 0695810 E 4960010 N (44°46′00.0311″, − 72°31′32.2070″); site (2) UTM NAD 83 Zone 18 T 0695720 E 4960030 N (44°46′00.7673″, − 72°31′36.2699″); site (3) UTM NAD 83 Zone 18 T 0695650 E 4960080 N (44°46′02.4551″, − 72°31′39.3823″)] on August 6 and 7, 2012. Elevation of the three sites was recorded at 369, 367, and 365 m , respectively.

### 2.2 Sample collection and processing

A portable pumping system was designed for a 16 ft Jon boat allowing us to collect 15–20 L of water aseptically from the epilimnion (0.5 m), metalimnion (3.5 m) and hypolimnion (25 m) of the pond at sites 1–3. The following notation was used for naming samples: epilimnion (1-S, 2-S, 3-S), metalimnion (1-M, 2-M, 3-M), and hypolimnion (1-B, 2-B, 3-B). Water samples were collected using two methods. On August 6, 2012, 15 L of water were pumped from each depth at site 1 and directly filtered through 0.45 μm polycarbonate filters (MilliporeSigma, Billerica, MA, USA). Filters were stored on ice and upon return to the laboratory maintained at -80^o^ C until processing for DNA extraction. On August 7, 2012, sites 2 and 3 within the pit pond were sampled. Water was pumped from each depth and collected aseptically in Whirl-pak bags (Nasco, Fort Atkinson, WI, USA) for the metalimnion and hypolimnion samples and sterile 5 gal polycarbonate containers for the epilimnion samples. Whirl-pak bags were stored on ice until return to the laboratory, and the sealed, polycarbonate containers remained submerged until returning to shore. These samples stored in Whirlpak bags were stored at 4^o^ C upon return to the laboratory. Within 12–24 h of collection, microorganisms from 10 to 15 L of water per site (2-S, 2-M, 2-B, 3-S, 3-M and 3-B) were concentrated onto 0.45 μm polycarbonate filters and stored at -80^o^ C for subsequent DNA extraction and Illumina library preparation at the Advanced Genome Technologies Core (AGTC) at the University of Vermont (UVM).

Physical and chemical parameters (temperature, pH, dissolved oxygen, specific conductivity, and light intensity) of the water column were measured using a Hydrolab DS5 multiprobe surveyor (Hach Hydromet, Loveland, Colorado, USA) and LI-COR LI-250A light meter with LI-192 quantum type sensor (LI-COR, Lincoln, NE, USA). Geographical coordinates were recorded with a Garmin Etrex High Sensitivity GPS (UTM NAD 83; Hazen's Notch USGS topographic map 2015 7.5 min series). Metal analysis of the water samples was conducted by the Agriculture & Environmental Testing Laboratory at UVM.

### 2.3 DNA extraction

Total genomic DNA was extracted from all nine samples using standard techniques at the Advanced Genomic Technologies Core at UVM. Briefly, 5 mL of sterile molecular grade phosphate-buffered saline (PBS) was added to the membrane filters in 50 mL conical tubes and shredded using a FastPrep-24 (MP Biomedical, Inc., Santa Ana, CA, USA) and a scalpel. The PBS was transferred to a new tube, centrifuged, and volume adjusted to < 0.5 mL. Homogenization was performed using a beater bead approach with the FastPrep-24 system and a 3 mm ceramic ball and AlO_3_ abrasive added to the samples. A cocktail of enzymes (10 μL of 10 μg/μL lysozyme, 4uL of 400 U/μL achromopeptidase and 2 μL of 5 U/μL mutanolysin) was added to the sample and allowed to incubate overnight at 37 °C. DNA was then extracted using the standard method outlined in the E.Z.N.A.® Mollusc DNA isolation kit (Omega-Biotek, Inc., Norcross, GA, USA), and the resulting DNA was quantified and assessed using the Nanodrop spectrophotometer (Thermo Scientific, Madison WI, USA), and Qubit Spectrofluorometer (Life Technologies, Carlsbad, CA, USA). Fragmentation of 10–100 ng of the resulting DNA was performed using a Covaris S2 AFA sonicator (Covaris Corp., Woburn, MA, USA) equipped with MicroVails to yield a size range of 200–500 base pairs as confirmed through a high sensitivity DNA chip on the Bioanalyzer 2100 (Agilent Technologies, Santa Clara, CA, USA). No genomic DNA was recovered from sample 1-B, and it was not included in further preparations.

### 2.4 Illumina library preparation

Library preparation was performed using 10–100 ng of DNA per sample in accordance with the Illumina TruSeq (Illumina Inc., San Diego, CA, USA) DNA Sample Prep LT version 2 SOP (Part # 15026486 Rev. C July 2012) with the indicated reagents (DNA kit #FC-121-2001) at the AGTC at UVM. Each sample was subject to end repair, adenylation, and ligation of Illumina adaptors for indexing purposes. PCR amplification was performed using Illumina reagents (Part #15012995) followed by quantification using the Qubit spectrofluorometer and qPCR quantitation (kit #4824, KAPA Biosciences, Woburn, MA, USA). Library quality and size distribution was assessed using the Agilent Bioanalyzer 2100.

### 2.5 Whole metagenome shotgun sequencing

Cluster generation and paired-end sequencing were performed using an eight lane high-capacity v3 flow cell on the Illumina cBOT and HiSeq 2000 sequencer, respectively, at the DNA Sequencing & Genotyping Center located in the Delaware Biotechnology Institute, an interdisciplinary research unit at the University of Delaware. Illumina PhiX Control v3 was used as a low-concentration spike-in during sequencing. Eight FASTQ files with raw sequence data were were delivered as one uncompressed tar file download.

### 2.6 Sequence processing

Bioinformatics infrastructure and analysis were provided by the Vermont Genetics Network (VGN) Bioinformatics Core. Raw sequences were examined for quality with FastQC v0.11.2 [3]. Adapters were removed and low-quality base calls and reads were filtered using Trimmomatic v0.33 [4]. A custom adapter file containing TruSeq universal and index primers, as well as the reverse complement of each, was used for removing adapters. Leading and trailing bases below quality 20 were removed. Additionally, reads were scanned using a 5-base wide sliding window and cut when the average quality per base dropped below 20. Reads < 75 bases in length were also removed (Trimmomatic parameters: PE –phred33 ILLUMINACLIP:TruSeq3_jjv5.fa:2:30:7 LEADING:20 TRAILING:20 SLIDINGWINDOW:5:20 MINLEN:75). Quality-trimmed FASTQ files for each sample were aligned to the PhiX genome (NCBI RefSeq NC_001422.1) using Bowtie2 2.2.3 [5] and all aligned reads were removed. Quality-trimmed and filtered reads were verified with FastQC prior to characterizing the taxonomic composition of the mine microbial community.

### 2.7 Taxonomic classification

Translated trimmed paired-end reads treated as single-end reads served as input for a protein-level homology search against NCBI-NR, a comprehensive non-redundant protein database (downloaded March 25, 2015), using the BLAST-like tool RAPSearch2 v2.16 [6]. All reads with alignments to the NR protein database, maximum 50 alignments per read, were imported into MEtaGenome ANalyzer (MEGAN) v5.7.10 [7] and parsed using a lowest common ancestor (LCA) algorithm. The following MEGAN parameters were used: maxMatches = 100 minScore = 50.0 maxExpected = 1.0 topPercent = 10.0 minSupportPercent = 0.5 minSupport = 50 minComplexity = 0.44 useMinimalCoverageHeuristic = false useSeed = true useCOG = true useKegg = true paired = false useIdentityFilter = false textStoragePolicy = InRMAZ blastFormat = BlastTAB mapping = ‘Taxonomy:BUILT_IN = true, Taxonomy:GI_MAP = true, SEED:GI_MAP = true, KEGG:GI_MAP = true, COG:GI_MAP = true. If sequences were unambiguously assigned to a taxon and passed the filter defined by these parameters, input reads were given a taxonomic assignment by LCA based on GI accession numbers and the complete NCBI taxonomy (ftp://ftp.ncbi.nlm.nih.gov/pub/taxonomy/taxdmp.zip downloaded Mar. 24, 2015). A single MEGAN combined sample file was generated from all individual sample MEGAN files with read counts normalized to the sample with the fewest input reads.

Two positive controls were used to validate our bioinformatics pipeline and to establish a minimum support threshold for taxonomic profiling. One control data set was comprised of single-end Illumina reads from a synthetic microbial sample prepared by CosmosID, which simulates organisms found in the Delaware River. A description of this constructed freshwater sample can be found here: http://www.cosmosid.net/constructed-freshwater.php. The second control dataset was a set of single-end Illumina reads from the Human Microbiome Project (HMP) mock community even sample (SRA accession SRR172902). Reads from both positive control samples can be downloaded from BaseSpace with a free account: https://basespace.illumina.com/projects/20039022/samples.

### 2.8 Statistical analysis

Statistical Analysis of Metagenomic Profiles (STAMP) software v2.1.340 [8] was used to test statistical significance of differentially abundant taxonomic groups and functional categories for each pit pond depth. LCA taxonomic profiles at all ranks including abundances, were imported to STAMP and a one-way ANOVA was used to compare strata with an effect size (ETA-squared) and multiple test correction using the Benjamini-Hochberg FDR method. Tukey-Kramer post-hoc test (0.95) was used to determine which means were significantly different when an ANOVA produced a significant *p*-value.

## 3 Results

### 3.1 Water chemistry

**Fig. 2:**
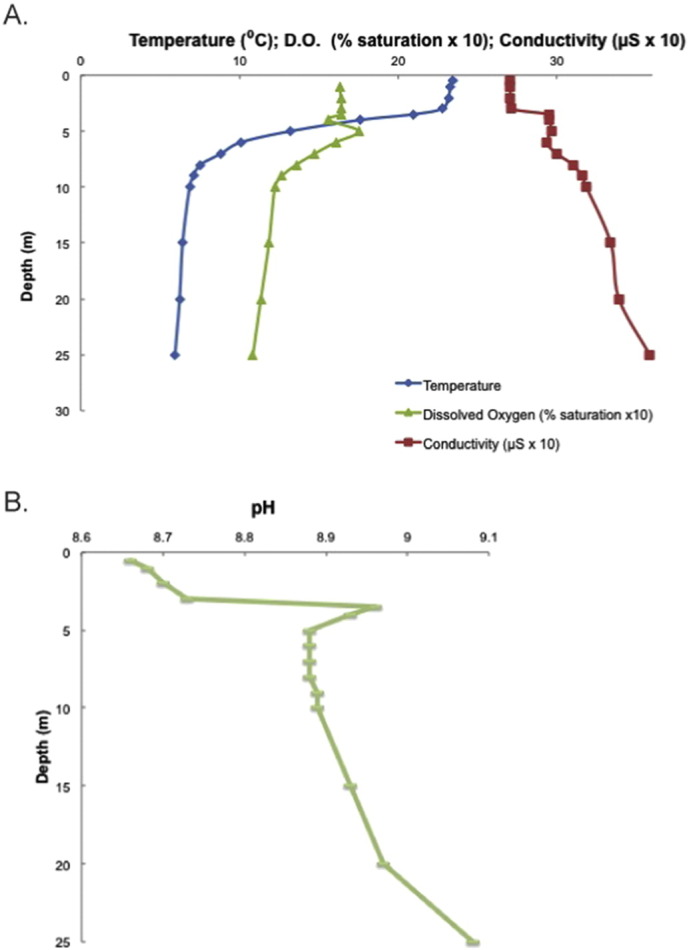
Environmental parameters: A) average temperature, dissolved oxygen, and conductivity B) average pH (Abbreviations: m = meters, ^o^C = degrees Celsius, μS = microsiemens, D.O. = dissolved oxygen).

Metagenomes were generated from samples taken at three depths along the water column at three distinct sites within the mine's pit pond. Environmental parameters of the epilimnion and metalimnion were notably different from the parameters of the hypolimnion. Marked differences were observed in pH, temperature, conductivity, light intensity, and concentrations of D.O. (Fig. 2, Supplementary Table ST1). The pit pond is alkaline, and in contrast to most freshwater lakes, pH increased with depth steadily rising from an average of 8.76 at the surface to 9.18 in the hypolimnion. Conductivity was high throughout the water table and increased with depth at all three sites. Average conductivity at the surface measured 271.3 uS increasing to 351.6 uS at 25 m indicating significantly higher ionic concentrations than expected in Vermont waters at this elevation (Vermont Department of Conservation, personal communication). The hypolimnion also presented greater extremes in temperature and light. Water temperature was nearly fourfold higher at the surface (23.53 °C) than in the hypolimnion (6.03 °C), and light was absent below 10 m. The steep thermocline revealed a narrow metalimnion. Oxygen levels remained near saturation throughout the water column and supersaturated in the thermocline. Heavy metal analysis of the water column revealed significant concentrations of Ni (26 5 μg/L), Fe (563 5 μg/L), and Mn (950 5 μg/L) in sample 1-B only. Levels of Ca, Mg, K, Na, Al, Fe, Mn, Cu, Zn and As were below 5 μg/L in all water samples (not shown).

### 3.2 Bacterial community structure

WMS sequencing of eight samples generated over 132 million paired-end reads, 101 bp in length, with an average depth of 16.5 million reads per sample (6–33 million) (Table 1). Seventy-one percent of raw reads (93,988,834) were retained after quality-trimming and aligned to the NCBI-NR protein database. Of the 48,646,975 reads with at least one hit to NR proteins, approximately 73% were assigned taxonomy by the LCA algorithm in MEGAN. Almost half of quality-trimmed reads in our samples (48.2%) had no protein hits in NCBI-NR and 27.2% of reads with protein hits could not be classified by the LCA algorithm. As a result, these reads were designated “Not Assigned” in MEGAN. There were 55,787 reads with repetitive sequence and were assigned to a “Low Complexity” node. The minimum-support percent threshold in MEGAN was set to 0.5% based on our bioinformatics workflow results from the HMP and Delaware River positive controls (Supplementary Figures SF1-SF2).

**Fig. 3:**
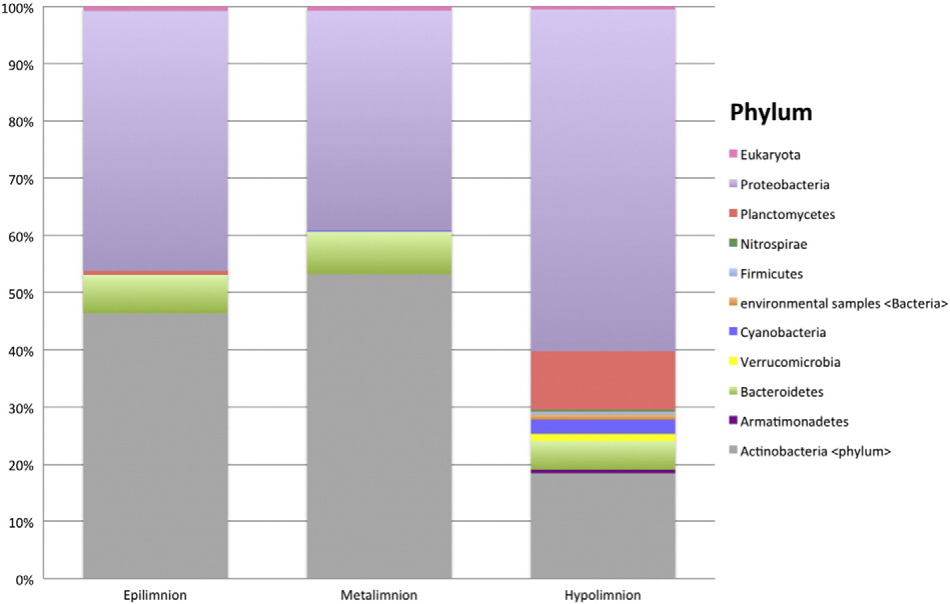
Percent relative abundance of reads assigned to each stratum at the phylum level by MEGAN (Strata: epilimnion = 0.5 m, metalimnion = 3.5 m, hypolimnion = 25 m).

The epilimnion and metalimnion exhibited a similar distribution of taxa at the phylum level (Fig. 3; Supplementary Table ST2), and were dominated by Actinobacteria (46% and 53% of reads, respectively), Proteobacteria (45% and 38%, respectively), and Bacteroidetes (7% in both). The phyla Cyanobacteria and Planctomycetes contained < 1% of the reads and each were present at only one site. The hypolimnion exhibited greater diversity of phyla and the distribution of reads differed from the surface and metalimnion. Within the two samples of the hypolimnion, nine distinct phyla were identified. These phyla largely paralleled the epilimnion and metalimnion, but a distinct distribution was evident. Proteobacteria was the predominant phylum containing 60% of the reads with markedly lower proportion of Actinobacteria (18%), Planctomycetes, (10%), Bacteroidetes (5%) and Cyanobacteria (2.5%). Armatimonadetes, Verrucomicrobia, Firmicutes, Nitrospirae, and unnamed phyla contributed < 1% each of the total reads, and with the exception of Verrucomicrobia, were exclusive to one sample. Read counts for phyla and other taxonomic ranks down to species are available for all samples in Supplementary Material (Supplementary Tables ST2-ST7).

**Fig. 4:**
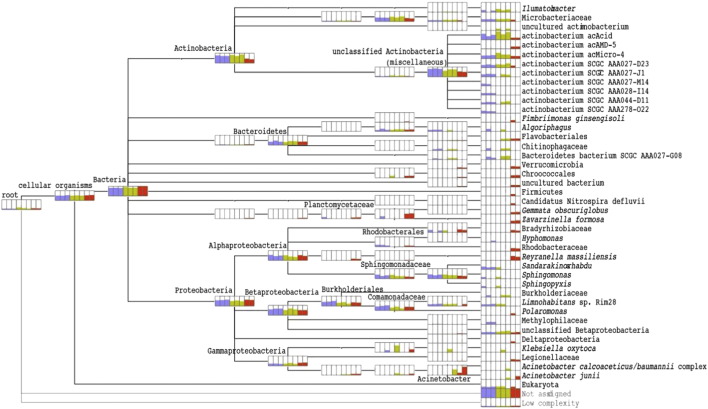
Taxonomic comparison of eight pit pond samples within three strata (blue: epilimnion, yellow: metalimnion, red: hypolimnion). Each node in the NCBI taxonomy is shown as a bar chart indicating the number of normalized reads from each sample that have been assigned to the node. Terminal taxa are the lowest common ancestor for each lineage shown, i.e. species, genera, or other ranks.

**Fig. 5:**
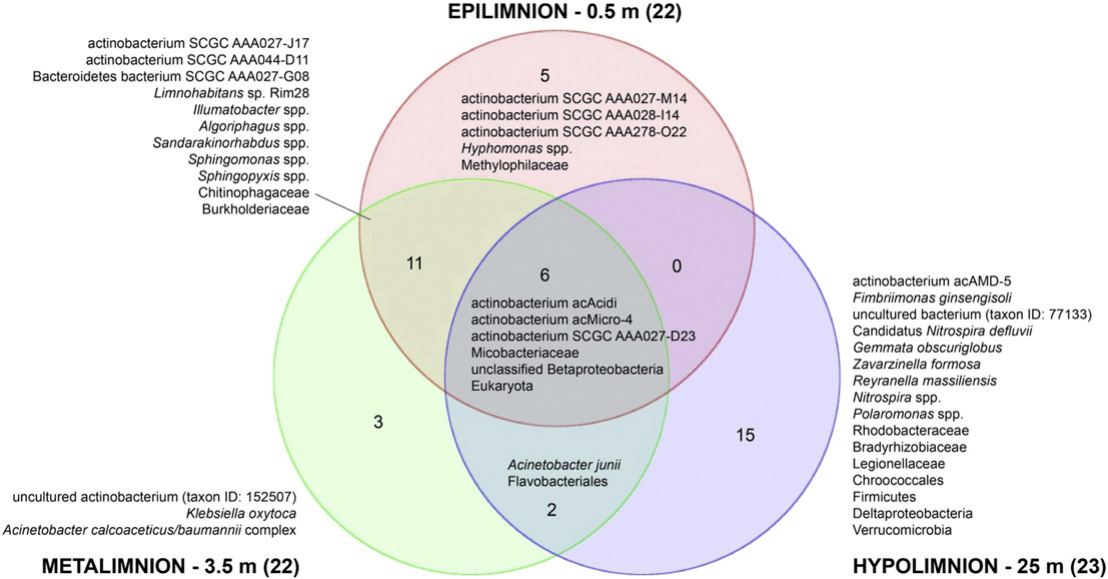
Venn diagram of lowest common ancestor (LCA) taxonomic assignments for epilimnion, metalimnion, and hypolimnion metagenomes constructed using Partek® Genomics Suite® software, version 6.6 build 6.15.1016, copyright 2014 (Partek Inc., St. Louis, MO, USA). Numbers in parentheses represent the number of LCA taxa assigned to each stratum. Numbers within the diagram represent taxa unique to each depth as well as taxa shared between depths.

Based on the LCA of each lineage in the NCBI taxonomy, we observed a set of taxa that were shared across the water column and others that were unique to each stratum (Fig. 4, Fig. 5; Supplementary Table ST8). The more highly alkaline, psychrophilic hypolimnion exhibited the greatest diversity with 15 LCA taxa representing nine phyla, including Verrucomicrobia, Firmicutes, and Nitrospirae. Taxa unique to the epilimnion and metalimnion belong to Proteobacteria and Actinobacteria. No taxon was shared between only the epilimnion and hypolimnion, but six occurred in all three strata, including three unclassified Actinobacteria. The surface and metalimnion shared 11 taxa, including the planktonic genus *Limnohabitans*, and the chlorophyll a containing genus *Sandarakinorhabdus.*

### 3.3 Statistical results

Results from STAMP showed significant differences with strong effect sizes at all taxonomic ranks (Supplementary Tables ST9-ST14). At the rank of phylum, the relative abundance of reads from Planctomycetes, Verrucomicrobia, and Cyanobacteria were significantly greater in hypolimnion samples than in epilimnion and metalimnion samples (with FDR corrected *p*-values of 7.98 × 10–6, 0.0002, and 0.0010, respectively). The relative abundance of reads from Actinobacteria were significantly lower in hypolimnion samples than in epilimnion and metalimnion samples (FDR corrected *p*-value of 0.007).

## 4 Conclusions

The distribution of microbial diversity is reflective of the VAG Mine pit pond's physical and chemical stratification. The epilimnion and metalimnion share a greater number of taxa (11 LCA taxa) than do the metalimnion and hypolimnion (two LCA taxa). The physiochemical properties of the upper two limnetic layers more closely paralleled each other and were in contrast to the hypolimnion, where inhabitants are required to metabolize at a higher pH, lower temperatures, and in the absence of sunlight. The genera *Limnohabitans*, *Illumatobacter*, *Sphingomonas*, and *Sandarakinorhabdus*, present in both the epi- and metalimnion, are ubiquitous freshwater plankton. *Sandarakinorhabdusis* is a novel, bacteriochlorophyll a containing, aerobic anoxygenic photoheterotrophic genus and *Sphingomonas* taxa are capable of producing the yellow carotenoid, nostoxanthin. Neither of these two genera were identified in the light-deprived hypolimnion. The contrasting environments of the metalimnion and hypolimnion are reflected by the paucity of shared taxa. Only two LCA taxa, *Acinetobacter junii* and Flavobacteriales were shared between these strata. No taxon was common to only the epilimnion and hypolimnion, but six LCA taxa were prevalent throughout the water column, including three unclassified Actinobacteria. It is noteworthy that over half of quality-trimmed reads in our samples (48.2%) had no protein hits. This is 30% higher than would be expected based on the proportion of non protein-coding regions in microbial genomes (approximately 20%), which should not align to proteins in the NCBI-NR database. Additionally, 27.2% of reads with protein hits were not given a taxonomic assignment by LCA and were designated “Not Assigned” in MEGAN. These results suggest the presence of novel proteins and/or organisms in this microbial community and that additional research and alternative bioinformatics approaches may help further characterize the metagenomes of the VAG Mine pit pond.

### 4.1 Nucleotide sequence accession numbers

Raw WMS Illumina sequence data from this study were submitted to the NCBI SRA under accession numbers SRS872561 (1-S: site 1, 0.5 m), SRS962537 (2-S: site 2, 0.5 m), SRS963313 (3-S: site 3, 0.5 m), SRS963552 (1-M: site 1, 3.5 m), SRS963574 (2-M: site 2, 3.5 m), SRS963594 (3-M: site 3, 3.5 m), SRS963611 (2-B: site 2, 25 m), and SRS963627 (3-B: site 3, 25 m).

## Conflict of interest

The authors declare no conflict of interest.

## Figures and Tables

**Table 1:** WMS sequence processing details per sample.

Sample name	1-S	1-M	2-S	2-M	2-B	3-S	3-M	3-B
Depth in meters	0.5	3.5	0.5	3.5	25	0.5	3.5	25
SRA Accession	SRS872561	SRS963552	SRS962537	SRS963574	SRS963611	SRS963313	SRS963594	SRS963627
Raw read count	6,159,936	9,104,554	9,615,156	12,487,606	15,069,910	25,203,526	21,538,064	33,113,412
Filtered read count	2,068,230	3,626,846	6,053,740	7,868,458	9,981,522	21,504,972	14,106,240	28,778,826
Filtered read length	75–101	75–101	75–101	75–101	75–101	75–101	75–101	75–101
Mean GC content (%)	53	54	50	50	52	53	49	49
Count of filtered reads aligned to NR	1,203,027	1,826,857	3,521,799	4,265,142	4,283,672	11,941,700	8,182,680	13,422,098
Count of aligned reads assigned taxonomy	893,334	1,319,058	2,639,892	3,256,959	2,847,347	8,926,747	6,172,087	9,349,475
Not assigned read count	308,393	506,035	878,543	1,003,465	1,431,030	3,002,889	2,001,672	4,054,262
Low complexity	1300	1764	3364	4718	5295	12,064	8921	18,361

## Appendix A Supplementary data

Phylograms depicting the lowest common ancestor taxonomic profile for Human Microbiome Project (HMP) control sample 902 (SF1) and CosmidID’s constructed freshwater sample from the Delaware River (SF2) generated in MEGAN 5.7.10 software [7]. The number of reads associated with each taxonomic classification is shown. The minimum-support percent threshold in MEGAN analyses was set to 0.5%. Image 2

Ecological metadata (ST1) and normalized read counts for each taxonomic rank (ST2-8) based on lowest common ancestor results from MEGAN v5.7.10 [7]. The minimum-support percent threshold in MEGAN analyses was set to 0.5%. Image 3

Results from Statistical Analysis of Metagenomic Profiles (STAMP) v2.1.340 [8] analysis to test the statistical significance of differentially abundant taxonomic groups for each VAG Mine pit pond stratum. Image 4
